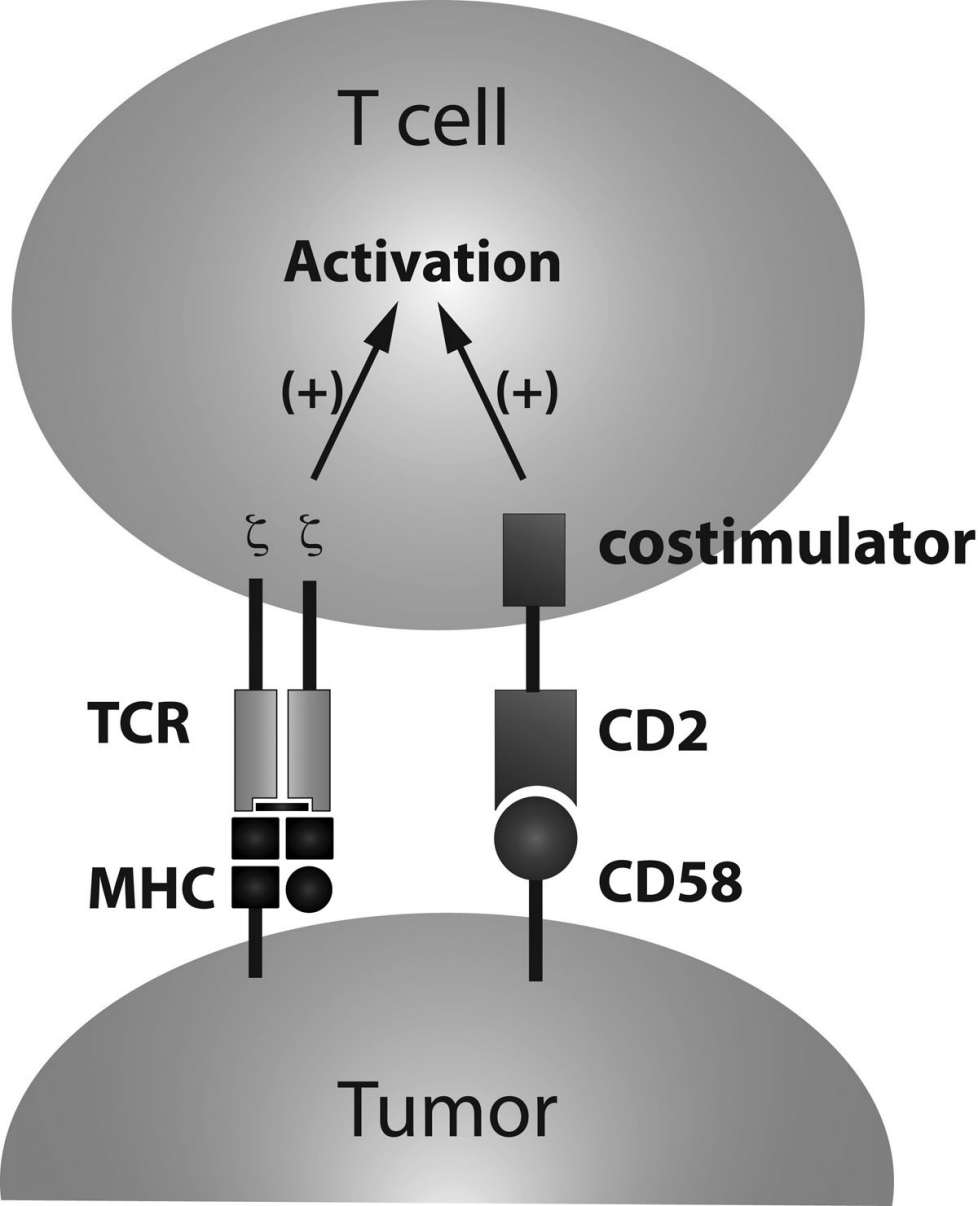# Augmenting adoptive T cell therapy through universal chimeric costimulators

**DOI:** 10.1186/2051-1426-1-S1-P14

**Published:** 2013-11-07

**Authors:** Ken-ichi Hanada, Prachi Bagadia, Qiong Wang, Kayla Griffith, James Yang

**Affiliations:** 1 Surgery Branch, NCI/NIH, Bethesda, MD, USA

## 

Adoptive transfer of Tumor Infiltrating Lymphocytes (TIL), T-cell receptor (TCR)-transduced PBL and Chimeric Antigen Receptor (CAR)-transduced PBL have proven to be clinically effective. CARs have evolved by introducing cytoplastic costimulatory domains. Higher cytokine secretion and longer persistence of CAR-transduced T-cells in vivo imply important roles of costimulatory signals. In contrast, costimulation of TIL or TCR-transduced PBL in vivo relies on the expression of costimulatory receptors on T-cells and ligands on tumor cells or antigen presenting cells (APCs). One way to give T-cells their desired costimulation signal independent of the endogenous receptor/ligand expression would be to introduce a fusion molecule that is comprised of a receptor whose ligand is frequently expressed on tumor cells and an intra-cellular costimulatory domain. In a pursuit for a “universal costimulator” that is applicable to various tumors, we produced fusions of CD2 and various costimulators such as CD28, 4-1BB, ICOS, CD27 and OX-40 (Figure 1). CD2 is the receptor for CD58 that is expressed on various tumor cells. Since CD2-CD58 complex localizes to the Supramolecular Activation Complex (SMAC) on activation of TCR, CD2-costimulators may signal in a TCR-activation dependent manner. Human PBL were retrovirally transduced with genes that encode NY-ESO-1-reactive TCR and each CD2-costimulator. In vitro co-culture assay showed higher IFN-γ and IL-2 secretion by CD2-CD28 and higher IFN-γ by CD2-OX40. To examine the in vivo effect of these fusion costimulators, vascularized SK23 melanoma tumor was established in NOD scid gamma (NSG) immunodeficient mice and human CD8+ T-cells transduced with anti-NY-ESO1 TCR and each fusion costimulator were injected. Out of five costimulators tested, CD2-CD28 showed a significant enhancement of anti-tumor effect. These results suggest that the CD2-CD28 costimulator may improve TIL and TCR-transduced PBL therapy.

**Figure 1:**